# Limits of the Rockefeller Foundation fellowship funding: Maria Palmira Macedo Tito de Morais and international nursing, 1936-1966

**DOI:** 10.1590/S0104-59702023000100046

**Published:** 2023-10-09

**Authors:** Ricardo dos Santos Batista

**Affiliations:** i Professor, Programa de Pós-graduação em História/Universidade do Estado da Bahia. Alagoinhas – BA – Brasil kadobatista@hotmail.com

**Keywords:** history of nursing, scientific trajectory, Portuguese Estado Novo, Rockefeller Foundation, Maria Palmira Macedo Tito de Morais (1912-2003, história da enfermagem, trajetória científica, Estado Novo português, Fundação Rockefeller, Maria Palmira Macedo Tito de Morais (1912-2003

## Abstract

The article analyses Maria Palmira Macedo Tito de Morais’ international nursing education as a Rockefeller Foundation fellow, during the Portuguese Estado Novo. It studies the local contexts influence on the international philanthropic agency’s actions, culminating in disputes with World Health Organization over the Portuguese nurse as staff. The sources are two dossiers on Maria Tito de Morais and her two fellowship cards collected at the Rockefeller Archive Center, a report of the Directorate-General of Health of Portugal and the journal *A Tribuna*, consulted at the Brazilian Digital Library. In conclusion, the Rockefeller Foundation’s strategies, when funding Maria Tito de Morais’ education aiming to hire her did not guarantee control over her professional trajectory.

## Resumo

Este artigo analisa a formação internacional de Maria Palmira Macedo Tito de Morais, como bolsista da Fundação Rockefeller no campo da enfermagem, durante o Estado Novo português. Compreende-se de que forma os contextos locais influenciaram as ações da agência filantrópica internacional, o que culminou em disputas com a Organização Mundial da Saúde pela presença da enfermeira portuguesa em seus quadros. São utilizados como fonte dois dossiês sobre Maria Tito de Morais e seus dois cartões de bolsista, coletados no Rockefeller Archive Center, um relatório da Direção Geral de Saúde de Portugal e o periódico *A Tribuna*, consultado na Hemeroteca Digital brasileira. Conclui-se que as estratégias utilizadas pela Fundação Rockefeller, ao financiar a formação de Maria Tito de Morais com o intuito de cooptá-la para seu *staff*, não garantiram o controle sobre sua trajetória profissional.

This article analyses the international education trajectory of the nurse Maria Palmira Macedo Tito de Morais during the Portuguese Estado Novo (New State). She was fellowship holder of the Rockefeller Foundation in two moments: from 1936 to 1939, with the expansion of education funding for Europeans in North America, and from 1958 to 1961, when the international philanthropic agency, previously aimed at public health, favoured the education of professionals in the field of biomedical sciences. The investigation is oriented by the following question: How does the trajectory of Maria Tito de Morais demonstrate fragilities in the Rockefeller Foundation’s actions in an international perspective?

The analysis of trajectories and/or biographies is important to understand tensions between the individual and the collective. According to Schmidt (2004, p.22-23), there are always points of contact between the experiences and conceptions of individuals and those of their contemporaries, because to a greater or lesser extent, all share certain cultural codes that enable coexistence and communication. Making visible the trajectories of nurses can function as a path for the construction of the professional identity in nursing (Padilha, Nelson, Borenstein, 2011); help the understanding of the elaboration of nurses’ symbolic capital in conducting their activities (Santos et al., 2018); and, among numerous other possibilities, reveal the protagonism of women funded by international agencies, which redefined the social image present in education and training patterns proposed in the early stages of nursing (Campos, 2013).

The documental corpus used is composed of two dossiers on Maria Tito de Morais, two cards of fellowship holder, the report of the Directorate-General of Health of Portugal and newspaper reports. The methodology is based on the ideas of Carlo Ginzburg (2007) about the importance of signs. The analysis of the sources draw on the indiciary paradigm described by Ginzburg, in which the small details reveal aspects that are not evident in the broad composition of the document. Although the dossiers about Maria Tito de Morais could a priori indicate that she would be a standard fellowship holder who corresponded to the Rockefeller Foundation’s expectations, specific and not highlighted information point to a more complex interpretative possibility.

This work falls within studies that question the limits the Rockefeller Foundation’s action, such as the work organized by Schneider (2002), to whom perhaps the broader lesson to be apprehended about the complex changes in biomedical research after the 1920s is the limited capacity of the agencies’ funding to control or foresee the influence of their grants. According to the author, this proposition surprises because these institutions’ programs often involved countries and entire populations, besides researchers who usually had other funding sources (p.5), thus hindering the accomplishment of their plans.

The Rockefeller Foundation was created in 1913, resulting from the philanthropy of the millionaire John Rockefeller Jr., who accumulated wealth with oil extraction, refining and sale in the United States of America (Farley, 2004). The Foundation was present in different parts of the world, such as China, Italy, Great Britain, Africa and South America, with large amount of investment in Brazil. It created education institutions based on the Flexner Report, published in 1910, which criticized the excessive number of students, the lack of full-time professors, and pointed out other problems in medical training in the United States in the late nineteenth and early twentieth centuries (Marinho, 2013); it carried out campaigns for the eradication of infirmities such as hookworm disease, yellow fever and malaria (Cueto, 1995); and granted fellowships for professional training in public health, especially in education institutions supported by the Foundation (Batista, Ferreira, 2021). The international agency based its actions on “demonstration-effect” (it conducted a “model” experience that later would be managed and replicated by the beneficiary country) and on the formation of “key-elements,” multipliers of the knowledge acquired with its support. The Fellowship Program of the Rockefeller Foundation was the principal means used to train those agents. Its objective was to select outstanding individuals, who were promises in the areas of interest defined by the institution’s general program, and help to prepare them to contribute significantly in the future, with research, education or public health (History of the..., s.d.).

Among the students who benefited from the Rockefeller felllowships were the siblings Maria and Augusto Tito de Morais. The daughter of Carolina Loureiro de Macedo de Morais and Tito Augusto de Morais, Maria Tito de Morais was born in Lisbon, on June 24, 1912. The couple already had a son, Manuel Alfredo Tito de Morais, born on June 28, 1910, about three months after the beginning of the Republic in Portugal.^1^ And, on April 12, 1921, Augusto was born, in Lourenço Marques (currently Maputo, the capital of Mozambique). The scientific trajectory of Augusto, who was granted a fellowship to study in Harvard, has not been studied yet by historiography.

The end of the Portuguese monarchy had the active participation of Admiral Tito de Morais, the family’s patriarch, who, on October 5, 1910, commanded the cruiser São Rafael and bombarded the Necessidades Palace (Palácio das Necessidades), pushing the royal family to Mafra and from there to exile (Assembleia da República, 2010, p.6). He was a militant in the Republican Party (Partido Republicano) and held positions in the Portuguese Republic: deputy in the 1911 constituent, senator until 1913, minister of the Navy in 1919, captain of the Ports of India from 1913 to 1916 and Governor-General of India in 1926. He kept his anti-fascist activity until the end of his life. His children followed, from early on, the republican and democratic ideals, which had as consequence their exile (p.7).

Manuel Tito de Morais, the eldest, was engaged in the democratic struggle, transited through countries such as Italy, Algeria, Angola (where he engaged in the anti-colonialist movement), and was exiled in Brazil between 1961 and 1963. At the Portuguese Revolutionary Junta (Junta Revolucionária Portuguesa), the governing body of the Patriotic Front for National Liberation (Frente Patriótica de Libertação Nacional) (created by democrats of various movements and the Communist Party), Manuel represented the Republican Socialist Resistance (Resistência Republicana Socialista), which he helped to transform into Portuguese Socialist Action (Ação Socialista Portuguesa), in 1964 (Assembleia da República, 2010, p.8-10), as resistance to the Estado Novo.

Under the control of António de Oliveira Salazar, the Estado Novo started in Portugal in 1933 and lasted until April 25, 1974, coming to an end with the Carnation Revolution (Revolução dos Cravos). According to Rampinelli (2014, p.120-123), the Salazarism was an authoritarian regime that gained stability due to internal and external reasons. In the internal context, Salazar used an economic, political and social strategy that consisted of the defense of a traditional rural world, with an illiterate population for its most part, opposed to any sort of land and agricultural modernizing reform. There was an internal dominant class characterized by its global economic debility, its external dependence, its division and the lack of sectors that would become hegemonic in processes of conservation or change. Finally, the social debility demonstrated the lack of the class entities’ politics, especially of the working class, due to the absence of industrial development.

Among the external factors was the fact that the regime had been implemented in a poorly industrialized country, whose main production was based on traditional agriculture, resistant to modernization. Lisbon was a colonialist metropolis, strengthened by the politics adopted by Salazar, characterized by extreme nationalism and administrative verticality. Lastly, Salazar revalorized his government’s position in the Spanish Civil War, maintained a false neutrality during the Second World War, obtaining economic advantages such as the maintenance of the regime and the territorial integrity of the colonial empire, in search of support from the democratic governments to the Estado Novo, and then threatened by the antifascist movement.

Salazar’s regime persecuted people it accused of being communists. The participation of the Tito de Morais family in democratic and socialist movements (also accused of being communists) did not produce reprisals from the Rockefeller Foundation to Maria Tito de Morais, as opposed to what happened to Brazilian medical personalities.

Recent studies demonstrate restrictions from the Rockefeller Foundation as to granting fellowships to openly communist students, or just suspected of this political orientation. One example is presented in the analysis conducted by Hochman and Paiva (2020) of the trajectory of the parasitologist Samuel Barnsley Pessoa, whose professional practice was connected to communism in the context of the Brazilian Estado Novo and the outbreak of the Second World War. Moreover, the authors show how, in the period from 1946 to 1964, the connections between parasitology and communism unfolded in the context of the Cold War. Also worthy of note is the study by Paloma Porto (2021) on the dynamics of scientific funding policies at the Biological Institute of the Federal University of Minas Gerais (Instituto Biológico da Universidade Federal de Minas Gerais). The author discusses how the programs of the Divisions of Natural Sciences and of Medical Education and Public Health, which financed the training of medical schools’ professors in Latin America for biomedical sciences, attributed the power of veto to the Rockefeller’s field personnel regarding fellowship holders dedicated to some specific themes of study.

Besides this introduction, the text is divided in three other sections. The first one analyses the first trip of Maria Tito de Morais to the United States and the hindrances encountered by Portuguese women regarding the access to professional education. The following section shows the conflicts in the second formative trip, in the 1950s, and the Rockefeller Foundation’s frustrations about the impossibility to control the destiny of professionals that it supported financially. Lastly, the final considerations relate aspects of the trajectory under study.

## From Portugal to North America: the funding of nurses by the International Health Division

Education occupied an important place in Maria Tito de Morais’ life trajectory. She was aware that in order to become a professional it was necessary to break away with the limits imposed to most women from the context in which she lived. According to Almeida (2014, p.340, 343), in the traditional Portuguese nation of the first half of the twentieth century, female responsibilities should never transpose the household sphere, nor be an object of paid work. Teaching represented one of the few careers opened to women, even though there were conquests in other areas, such as nursing.

Maria Tito de Morais did not attend regular school beyond elementary education, actually a hindrance faced by some of her compatriots between the 1920s and the 1930s. Regarding schooling in Portugal, Candeias and Simões (1999) analysed the population censuses between 1900 and 1960 and affirmed that the access to written culture in the country was slow, in a doubly peripheral process in the European context: peripheral in relation to the “hard core” of literacy and, along the twentieth century, peripheral in relation to the limits South, East and West, which have been historically less permeated by written culture. While most of Europe registered a literacy rate of 98% in 1950, Portugal presented only 55%; in 1900, only 25% of the Portuguese population could read and write. Nevertheless, due to her family’s reasonable financial conditions, Maria Tito de Morais continued her studies at home and, besides the courses of music and domestic arts, she concluded the equivalent to the fifth grade of secondary school, with one more year of French and two of Portuguese (Hill, 13 abr. 1936).

Historically, nursing education in Portugal differed from other European countries for the absence of religious women. According to Silva (2012), the Hospitaller Order of Saint John of God (Ordem Hospitaleira de São João de Deus) (male) performed a crucial role in the care of the sick in Portugal since the seventeenth century, and the long tradition of male nursing was not ignored in the construction of the first schools of nurses education.

Among the institutions created during the nineteenth century, which were dedicated to nursing education, it is possible to identify the first course in the Hospitals of the University of Coimbra in 1881; and a school of male nurses in Lisbon in 1886 (both institutions had momentary operational interruptions). On the other hand, the Saint Antonio General Hospital (Hospital Geral de Santo António), administered by the Holy House of Mercy (Santa Casa de Misericórdia) of Porto, had nurses divided by gender: male nurses took care of male patients, and female nurses took care of women (Silva, 2012, p.20). In the city of Porto, like in Coimbra and Lisbon, the school accepted male and female students. However, the male tradition in nursing and the low level of female education prevented the access of women in the profession for a long time. The enrolment records at the School of Nursing (Escola de Enfermagem) in Porto indicate that in several moments, men corresponded to most of the enrolments. In 1921, for instance, the male gender represented 83% of the enrolments and more than 70% in three other years (Silva, 2012). The possibilities of professionalization in this field started in the 1930s, by sending young Portuguese women to the United States to study, financed by the Rockefeller Foundation. Upon their return, it was expected that they would act as knowledge multipliers in institutions, e.g., the Health Center in Lisbon (Centro de Saúde de Lisboa) and the Technical School of Nursing (Escola Técnica de Enfermagem), also in Lisbon, created respectively in 1939 and 1940.

In 1936, the young Portuguese woman had the idea and, indeed, convinced her father, Admiral Tito de Morais, to look for the physician José Alberto de Faria (Faria, s.d.),^2^ Director-General of Health, and ask the International Health Division (IDH)^3^ for a scholarship to study nursing in the United States. The Rockefeller Foundation staff was enthusiastic about the application interview. Being only 25 years old, she demonstrated to be serious and well prepared. George Strode,^4^ director of IHD in Europe and responsible for the selection process, analyzed five candidates, of whom three were selected: Jaime Pereira, for sanitary engineering; Maria Angélica Lima Basto and Maria Palmira Tito de Moraes, for nursing (Memo n.73, 5 maio 1936). At that time, Maria M. Monjardino, the first Portuguese student financed by the Rockefeller Foundation, was already dedicated to nursing in the United States.

In 1936, there were virtually no facilities for nurses’ training in Portugal. The sanitary education in the country started in 1901, with a law that created the Central Hygiene Institute (Instituto Central de Higiene). This education center, which had other functions, such as to promote works of practical hygiene, propagate the findings in the field of hygiene, and conduct laboratory analyses, was pedagogically linked to the School of Medicine of Lisbon (Faculdade de Medicina de Lisboa), in 1911, but under the Ministry of Home Affairs (Ministério do Interior) as part of the Directorate-General of Health (Diretoria Geral de Saúde), in 1929.

The international meetings on the role of the Schools of Hygiene in Europe demonstrate the importance they had for the professional education in health. The League of Nations met with directors of the schools of hygiene in May and July 1930, in Paris, France, and in Dresden, Germany, and concluded that this educational type was the best for the training of sanitary technicians, which should occur with the support of public and/or private institutions, according to local conditions (Faria, 1934, p.293-294). In the report prepared by Professor Prausnitz and approved at the Conference of Dresden, there was the discussion about the need of different categories to obtain training with deeper technical knowledge on hygiene, e.g., sanitary inspectors, “female nurses” and midwives (Faria, 1934, p.294).

At that moment, schools of hygiene were identified in the United States, in Brazil, but also in Warsaw, Budapest, London, Zagreb, Prague, Athens and Paris, with variable education duration and based on research work. In Poland, e.g., there were centers of sanitary demonstration at social hygiene dispensaries for the practical training of visiting sanitarians and nurses, of which the most important was in the district of Mokotów, in Warsaw. Aware of this picture and after the proposal presented by José Alberto de Faria to Salazar, who was still the minister of Finances in 1929, the Rockefeller Foundation decided to extend its support to Portugal. The institution had already financed the creation of schools of hygiene such as the Johns Hopkins, in Baltimore; the Harvard University, in Boston; besides institutions in Toronto, London, Prague, Warsaw, Budapest, Belgrade, Zagreb, São Paulo, Angora, Calcutta, as well as medicine education in Canada, United States, London, Brussels, Lyon, Hong-Kong and Singapore (Faria, 1934, p.298-299).

The physician Daniel O’Brien, member of the section of medical sciences at the Rockefeller Foundation, went by Portugal and organized a meeting between Faria and George Strode. Soon after, the Portuguese received a correspondence with the following determinations: (a) it was necessary to have an authorization from the Rockefeller Administrative Council to sign the cooperation with the Portuguese; (b) an investigation would be conducted *in loco* to decide how the agency would intervene in Portugal without violating the limits of its current program; (c) in the case of cooperation, the support would be initially modest, providing fellowships for a group of young people to improve their knowledge in hygiene; (d) the work would be developed by a specialized technical group, the staff should be full-time dedicated to the service, and the wages would be sufficiently high as to attract “best class” physicians and nurses, with no need to take other jobs (Faria, 1934, p.302).

When returning to the United States, on November 9, 1931, Strode communicated informally the approval of the cooperation and, on April 25, 1932, he visited Portugal to study the conditions of the support that could be provided. He arrived accompanied by the physician Rolla Hill, who would become the Foundation’s representative in Portugal and, in January 1933, he shared the following message: “The members of the International Health Division of the Rockefeller Foundation are authorized to officially inform the Government of Portugal that the Foundation accepts its invitation to collaborate within the limits that the scientific directors can, from time to time, approve” (Faria, 1934, p.307).

Based on this agreement, physicians and nurses were sent to the United States. The fellowships of Maria Tito de Morais and Maria Angélica Basto were approved for the period of one year, starting on September 1, 1936 (Sawer, 25 jun. 1936), and later extended until 1939. The situation of the two Portuguese fellows presented the specificity of women who wished to professionalize in health, but who never in their lives had any contact with this field. This was a different situation, e.g., from the Brazilians also financed by the international philanthropic agency. The creation of the Anna Nery School of Nursing (Escola de Enfermagem Anna Nery, EEAN), in Rio de Janeiro, drawing on the Parsons’ mission, in 1923, ensured practical experiences for the students in institutions such as Hospital Geral de Assistência, Hospital São Sebastião (infectious and contagious diseases), Hospital Arthur Bernardes (mother-child assistance) and Maternidade Pro Matre (Ferreira, Salles, 8 out. 2019). Therefore, they already were professional nurses.

Aiming to approach the sanitary field, the Portuguese candidates spent the summer of 1936 in the wards and surgery room of a hospital in Lisbon, visited the Ministry of Health to study its organization, together with Maria Monjardino, and visited sections to learn about the work of visiting nurses or social workers: “The Epidemics section, the Infant Welfare station, and the tuberculosis survey in Villa Franca were the principal places visited” (Hill, 20 ago. 1936, p.1). The staff of the Rockefeller wished to provide at least an idea of the Department of Health organization and the work it conducted, so that the fellows could critically judge similarities and divergences between what they saw in Portugal and what they would see in the United States.

The education of the Portuguese women in the United States started at the Western Reserve University. From the beginning, Maria Tito de Morais demonstrated lack of confidence regarding her own work, which occurred in the formative experience from 1958 to 1961. Parts of documents, such as “She has felt that she was not doing as well as she should, or as well as she is capable of doing, and thought that probably the teachers were not satisfied with her work,” or “was discouraged because she didn’t do better” (Memorandum by Dr. Hill, 23 jan. 1937), show the impressions of the Rockefeller about the expectations of a young woman around her own performance, exceedingly demanding of herself.

The “three Maries” incorporated themselves into the class of the school of nursing and became popular. The surname Tito rendered the nickname “Mosquito Tito” to Maria Tito de Morais, who was not annoyed and accepted calmly the North American provocations (From Miss…, 6 abr. 1937). Although she was not the favorite among the three, it is likely that she was the one who rose to greater prominence through her career.

An issue that marked the early years of training relates to the diploma of nurse. According to Rolla B. Hill (citado em Memo n.93, 1 set. 1938, p.1):

One of the few objections to the Cleveland School is that the girls have aways been treated as special students, and after spending two years there and doing all the required work – in the case of Miss Lima Basto and perhaps others, better than the majority of the class – have nothing to show for it except a letter from the Dean stating that they have spent some time at the School.

I understand that Miss Tito de Morais and Miss Lima Basto are to take the regular course in Toronto. Perhaps it will be possible for them to obtain an official certificate or diploma froom this School.

The Western Reserve University did not provide a certification to the foreign students. This issue was resolved by sending them to the School of Nursing at the University of Toronto, Canada. The diploma was considered an important achievement, especially when returning to Portugal, because it functioned as an element of professional distinction in a context in which few women had access to a similar education.

While the students were dedicated to nursing, the Rockefeller staff discussed their future directions in Portugal. The nurse Mary Beard questioned George Strode whether it would not be necessary for them to be closer when choosing the next fellowship students. At the same time, she indicated that Maria Basto seemed more mature than Monjardino and Tito de Morais, therefore she should have a more important role at the Technical School of Nursing of Lisbon (Escola Técnica de Enfermagem de Lisboa) (Beard, 25 fev. 1938). The Paris office, from where the Rockefeller actions in Europe were coordinated, proposed the creation of a school in a rented building, with a nurse, a secretary and a housekeeper. The other two nurses would be designated to the health center, where they would continue the nursing care at the tuberculosis dispensary. The main criticisms were about the possibility of instituting an improvised service, since the physician Francisco Gentil (Enquadramento Histórico, 15 jul. 2007, p.10)^5^ had already created a school project for which no support was obtained. Besides, it was not of question the idea to inaugurate a school in Coimbra, in association with the missionaries Franciscanos de Maria (Crowell, 26 mar. 1938).

Even before Maria Tito de Morais’ return to Portugal, which occurred earlier than planned, on October 4, 1939, because of the Second World War, the international philanthropic agency expected that Salazar’s government kept the promise to provide official jobs to the nurses, even if there was no guarantee about this. A correspondence between Rolla B. Hill and José Alberto de Faria discussed the problems to be faced by the first three nurses trained in the United States. Maria Monjardino was the daughter of the physician with the same surname, who had “Difficulty with the government over his appointment as assistant professor several years ago. He has just been ousted fro his post by the supreme court. It appears he is now persona non grata, and the non grata part may extend to this daughter” (Excerpt Dr. Faria, 27 jul. 1938, p.1). It is likely that doctor Monjardino’s nomination occurred during the Republic and this identified him as a defender of that regime and, consequently, an opponent of Salazarism. Maria Basto was the daughter of a former monarchic minister who had conflicts with the government too, which was also the case of Maria Tito de Morais’ father, whose performance in the Republic already has been mentioned (Excerpt Dr. Faria, 27 jul. 1938, p.1).

The local dynamics had strong potential to modify the plans of the Rockefeller Foundation, especially with the conflicts resulting from Salazar’s regime. However, the documentation presents a prominent professional role for Maria Tito de Morais upon her return to Portugal. She worked at the health center until October 7, 1940, when the Technical School of Nursing, created by the Decree-law n.30447, of May 17, 1940, was inaugurated in Lisbon and installed at Republic avenue, number 18 (Ferreira, 2012, p.103).

The professional’s full-time hiring by the School of Nursing was negotiated with the head for the Health Center of Lisbon, to which staff she belonged. The physician Almiro Maia de Loureiro, who had been an assistant to Francisco Gentil and fellowship holder of the Rockefeller, needed a nurse to develop the projects of the Center. He intended to cede her part-time only. On the other hand, Gentil demonstrated objections to her participation as a professor, possibly because her family belonged to an opponent group and he disapproved of their political ideas. However, in Portugal there was no other nursing professional with the necessary qualification to be a professor at the new education institution.

The transfer of Maria Tito de Morais to the School required changes on the program of public health nursing at the Center. The situation worsened when Maria Monjardino informed the intention to leave her job in January 1941, to get married, which forced the return of her colleague (Ferreira, 2012, p.144-145). While Monjardino interrupted her professional trajectory to fulfil the attributions socially expected from a woman, in the private and family sphere, Maria Tito de Morais was engaged in meeting a professional demand to which very few Portuguese women were qualified.

Between 1946 and 1950, she got back to studying, this time at the Faculty of Letters of Lisbon, receiving her undergraduate teaching degree in 1951 (Personal…, 1958). Being forbidden by the Salazar regime to work at the Health Center and the School of Nursing, Maria Tito de Morais left Lisbon. However, due to the international recognition of her performance, she was hired by the World Health Organization (WHO). The new job took her to work in Brazil and later to a second fellowship of the Rockefeller Foundation.

## Maria Palmira Tito de Morais and the frustrations of the Rockefeller Foundation in nursing

When Maria Tito de Morais received the Rockefeller Foundation’s proposal of a second fellowship, in the 1950s, she worked in Rio de Janeiro as a nursing consultant at the WHO/Pan American Sanitary Bureau and was concluding the “Survey on nursing resources and needs in Brazil (1956-1958)” (Levantamento de recursos e necessidades de enfermagem no Brasil (1956-1958)), coordinated by the nurse Haydeé Guanais Dourado, from Bahia. This research started to be planned at the seventh Brazilian Congress of Nursing (Congresso Brasileiro de Enfermagem), in 1954, in São Paulo, with the objective of achieving better knowledge on the country’s reality in that field, e.g., the number of nurses, their education and working conditions.

Maria Tito de Morais worked with Guanais Dourado, who was also a fellow of the Rockefeller Foundation twice. The research final report was delivered on October 30, 1958, with 46 recommendations to the Ministry of Education, the Ministry of Health and institutions that maintained nursing schools, among others (Malta, 2012, p.19-20, 94).

At that moment, Maria Tito de Morais was 46 years old and single, and had a broad professional experience. She worked at the Health Center of Lisbon and the Technical School of Nursing of Lisbon between approximately 1939 and 1949. She knew India, East and West of Portuguese Africa, South Africa, Middle East, Europe (Italy, Greece, Switzerland, France, England and Spain), United States and Canada, Central America and Brazil, mostly from the work developed at WHO. She was fluent in English, Portuguese and French, had a good knowledge of Italian and Spanish, and understood fairly well German and Arabic (Personal…, 1958). She advised the Syrian government on nursing issues and was a senior member of the Nursing Education team in Iran, until she was sent to Brazil. According to Marcolino Candau, President of WHO:

In all her assignments Miss Tito de Moraes has demonstrated real ability and has established excellent relationships with her colleagues and with the Governments. She has expressed interest in more senior nursing posts with the Organization and appreciates her need for further study in nursing in order to become better prepared for promotion (cited in Mcandau-Jcbugher, 17 abr. 1958, p.1).

Maria Tito de Morais expected to resume her activities at the multilateral agency at the end of her study period, continue to advise national and international nurses on planning and public health nursing services, and conduct teaching and research activities in the field of nursing. The Teachers College, of Columbia University, received her at the end of 1958. According to Ferreira and Salles (8 out. 2019), the institution was one of the chosen by the Rockefeller Foundation, as well as the Philadelphia General Hospital, at the School of Nursing of Toronto, to ensure professional distinction to fellow nurses and open the way for them to occupy positions of power in the bureaucratic structure and the conduction of schools of nursing in Brazil, such as the EEAN. In December 1957, approximately one year before beginning her studies, the candidate went on a trip to the United States and was encouraged by Virginia Arnold, assistant director at the Rockefeller Foundation, to talk to some members of Columbia University about her plans, to build closer professional relations. She gave a strong impression among the people she met (Arnold, 21 jan. 1958).

When analyzing the correspondence between Arnold and Robert Briggs Watson,^6^ it can be identified that, before the implementation of Maria Tito de Morais’ fellowship, there had already been interest from the Rockefeller to incorporate her into the staff. Virginia Arnold (21 jan. 1958, p.2) made explicit the reasons why the invitation could not be made yet:

In regard to MPTdeM’s possible RF employment, I have not pursued this point with JBC for two reasons: (1) I cannot yet justify a second nurse on the staff and (2) because the fellowship, MPTdeM woud not be available for another year … You know that I feel she would be a great asset to the RF nursing program.

The possibility of hiring Maria Tito de Morais was discussed on other moments. In October 1958, Robert Watson affirmed that she fulfilled the specifications for the job, but she was not a strong person. In the past two years, since he had met her, she became ill on several occasions. At the same time, he believed that the nurse did not make firm decisions, and mentioned the issue of the trip to the United States as an example to be considered (Excerpted..., 1 out. 1958).

About this last issue, exposed by Watson, initially there was an agreement that the trip would be by ship, and the ticket was reserved. Then, Tito de Morais thought that she would not be able to arrive on time, due to her appointments in Brazil, and cancelled the ticket without informing the Rockefeller staff (Excerpted..., 1 out. 1958). The nurse would have stated that she would have followed if she was assured to arrive one or two days after the expected, but when Watson tried to book another ticket, she did not succeed. The Rockefeller Foundation established a series of criteria and procedures since the beginning of the fellowships policy, but communication failures between members of the international agency offices and the fellowship holders were recurrent, as in this case. An example can be observed in the episode involving Euvaldo Diniz Gonçalves, from Bahia, in the 1920s. He married and did not communicate the Rockefeller Foundation. The information arrived only on the eve of his trip, implying an urgent change on the amount of his fellowship, since it was different when the spouse went along on the trip (Batista, 2020b, p.326).

Without a ticket to sail, Maria Tito de Morais travelled by airplane to the United States. Her arrival was delayed in five hours, due to a revolution that occurred in Venezuela, a process that she certainly approved. At the stopover in Caracas, soldiers armed with machine guns surrounded the aircraft and prevented its departure for some time (Arnold, 8 set. 1958). The dictatorship of Marcos Pérez Jiménez, implemented on June 13, 1953, was at the end. The New National Ideal (Nuevo Ideal Nacional) that he propagated was a modernizing project, with a strong anti-communist perspective, which pointed to the capitalist consolidation of the Venezuelan socio-economic structure (Rodríguez, 2011). That episode may have given hope to the nurse, who saw it from inside the aircraft, regarding the collapse of the Portuguese Estado Novo too.

Finally, Robert Watson questioned if there would be a moral commitment of the Rockefeller or even of Maria Tito de Morais with WHO, since the fellowship was destined to her permanence at the originating agency. Although he could wait until the end of her training to know if she would be the most appropriate candidate to the position, this would delay the development of the programme of nursing in Brazil (Excerpted..., 1 out. 1958).

In contrast to Watson, Virginia Arnold defended the performance of the Portuguese nurse. For her, Maria Tito de Morais was “superior,” because she had a richer education, greater experience, and cultural education, which was a benefit in the international field. She recognized the nurse had health problems in Brazil, but, after having met her in person, understood that it was not a hindrance. The humidity in Rio de Janeiro and the fact that she lived in a hotel (and therefore had no control over the preparation of her food) were aggravations for gastrointestinal issues. Besides, Arnold minimized Maria Tito de Morais’ indecision about the ship travel, since “in the area in which decisiveness is important, namely her job, she is quite clear in her thinking” (Excerpted, 24 out. 1958, p.1). Finally, she did not considered it polite with Marcolino Candau to give a fellowship to one of his staff and immediately offer her a job at the Rockefeller Foundation: “I believe that she also feels a responsibility to return to WHO for a least a year because of the fellowship” (p.1).

In June 20, 1958, the nurse’s grant was approved for a period not exceeding 12 months, and counted with the recommendation of members of the different international health agencies, such as Robert Watson and Virginia Arnold; Agnes W. Chagas, regional counsellor of Nursing at the Pan American Health Bureau, and Kenneth Courtney, of the same institution. Furthermore, Maria Tito de Morais was the third indication of Marcolino Candau for funding of the Rockefeller (Fellowship for…, 20 jun. 1958).

The nurse received a monthly allowance of 250 dollars and a pension for her family, in Portugal, dependent on her. The admiral Tito de Morais died on July 14, 1963, at 83 years old, according to the newspaper from São Paulo *A Tribuna* (Faleceu..., 14 jul. 1963, p.2), from a long-duration disease. This infirmity, associated to the disdain of the Salazar regime, which might have charge him with financial sanctions, may have influenced the condition of him being dependent on Maria Tito de Morais.

The posture of the Rockefeller Foundation put no emphasis on the political position of the nurse and her family. According to Rampinelli (2014, p.127-128), the colonial wars were responsible for the Salazar regime’s fall in Portugal. For this reason, the dictatorial government explored the international bipolar conjuncture using the thesis that communism would take over in case the metropolis withdrew from the Portuguese colonies. Manuel, Maria Tito de Morais’s brother, was a detainee at Aljube prison and, after being freed on bail, not being able to find a job in Portugal, went to Angola, where he was intensely involved in the anticolonial war (Assembleia da República, 2010, p.9). Although the United States official means adopted, due to the momentary convenience, an apparent policy of cooperation with the colonial nations, official documents testify that anti-colonialism grew in the country (Rampinelli, 2014, p.128).

The nurse Virginia Arnold seemed not to be aware of the political conflicts in which the Tito de Morais family was involved in the European context. She was troubled with the suspicion that the nurse did not wish to abdicate from the Portuguese citizenship to receive the North American and wondered about a possible “political” relation of Maria Tito de Morais with Portuguese friends. Nevertheless, Arnold was certain that, if a proposal was made, she would accept to work at the North American agency:

In the meantime, however, one point in the problem has become somewhat clearer in my mind. At the time of our conversation with JBC on MPTdeM employment, the question of US citizenship came up. At the time of our conversation with JBC, I was under the impression that MPTdeM did not wish to give up her Portuguese citizenship. As you know she is a very proud individual. However, I have now learned that she has recently discussed this point with her family and close friends in Portugal. I learned that her family is quite in accord with her wishes on this point but some of her friends (political?) are not because of her great strength and support (Arnold, 21 jan. 1958, p.2).

The “strength and support” emphasized by Maria Tito de Morais’ friends referred to the participation in the Movement for Democratic Unity (Movimento de Unidade Democrática). She was actively involved in the campaign for the presidential election of general Norton de Matos, opponent of Estado Novo, in 1949, and the protest against the imprisonment of democrats. For this reason, she was dismissed on the same year from the Health Center of Lisbon, and one year later she was prevented from assuming any position at the Technical School of Nursing, the Portuguese Institute of Oncology, and all State agencies (Associação…, s.d.). Although being away from her country, she maintained an expressive political action, resulting from the engagement of a family “symbol of the Republic,” image that could be weakened by the adoption of a new nationality, considered a treason to the country.

Seen in the funding institution as apparently free of associations with communism and living in the United States, Maria Tito de Morais started to attend University, in September 1958, and the wish to obtain the doctorate degree from the Teachers College contributed to the renewal of the fellowship for one more year, as from September 8, 1959. Between the end of 1958 and the beginning of 1959, she studied themes especially directed to tutoring in nursing, as can be seen on Table 1:

**Table 1:** : Disciplines followed by Maria Palmira Tito de Morais at the Teachers College

Subject	Professor
Education and Society	Gordon Lee
Social Psychology for Educators	G. Watson
Educational Research and Planning	Lorge
Professional Advanced Course (nursing problems)	R.L. MAcManus
Curricular problems in the pre-service (nursing education programs at higher education institutions)	M. Montague
Studies on nursing services and nursing education (advanced)	H. Bunge
Role and functions of consultation in nursing education	R. Gilbert

Source: M.P. Tito..., 18 dez. 1958.

The nurse devoted great efforts to obtain her doctorate degree. When applying for the program in Philosophy with specialization in Nursing Education Consultancy, she took the CAVD examination required for students who are non-native English speakers (Bunge, 14 dez. 1958). She was praised on several moments for her performance and workload. Differently from other doctorate candidates, the previous education in letters was advantageous in the liberal arts. Usually, nurses interested in becoming doctors of philosophy had a bachelor degree, with specialization in nursing and deficiency in the other area.

The Rockefeller nursing staff showed “great concern about WHO’s unwillingness or inability to put Maria Palmira Tito de Moraes in a position commensurate with her ability and skills” (Excerpt, 18 dez. 1959, p.1). She was considered an extraordinarily capable person, with talents not used by WHO to their fullest, and which could be better utilized if she would be incorporated by the North American philanthropic agency.

Maria Tito de Morais needed to enrol in courses until June 1960, which implied attending a maximum amount of credits until the end of 1959 and beginning of 1960. According to Hattie Jarmon (16 fev. 1959, p.1), admission official at the Teachers College, “I think I should state, however, that this would mean carrying a very heavy load, particularly with regard to the time involved in the research project and preparation of the report.” An alternative would be to conclude the disciplines of the course until June 1960, and the research project and the report after returning to her position at WHO. In this case, she would need to return to the university to present the final report and to take the oral exams.

However, she was not properly advised about the enrolment for that year’s second semester, and Virginia Arnold had to intervene. For the supervisor, the confusion was caused by a dispute between the Teachers College and Columbia University, and for this reason she stated that it was unpleasant “to get the mess strainghtened out” with all those involved (VA Diary, 3 fev. 1960, p.1). She considered that doctor G. Watson, the tutor of Maria Tito de Morais at Columbia University, was the main source of hindrance, even if unintentionally, since he was “one of those top-flight professors who couldn’t care less about university requirements, regulations etc. In fact, he admitted to me that he probably had not kept MPTdeM well enough informed on all the requeriments” (VA Diary, 3 fev. 1960, p.1).

This episode implied the reduction of the time limit to deliver the nurse’s final work, and she wrote a letter to Virginia Arnold assuming the responsibility for what had occurred. Among the issues addressed, she lamented for not having fulfilled all requirements to conclude her studies in June 1960, because she would not be able to finish the final report until February 24, which was the date informed for depositing the document, whereas she had hoped to finish it in April. Maria Tito de Morais demonstrated her commitment to the studies: she enrolled in the maximum number of credits allowed; she was approved in the exams of French and Spanish; and started to study German, when the university’s rules changed, requiring that there should be one Germanic and one Latinic language. Furthermore, because she had not been able to finish the draft of the work, she felt “extremely embarrased with this failure” (Morais, 10 fev. 1960, p.1-2).

She demonstrated not to have a perception of the conflict between the Teachers College and Columbia University, besides apparently not suspecting how much the Rockefeller Foundation wished to have her. She repeatedly apologized, because in her imagination she did not correspond to the expectations deposited on her. Arnold tried to de-construct this perception:

As I am familiar with the difficulties you have encountered, I hope that you will not feel it is due to your inability to carry doctoral level work. On the contrary, you have already demonstrated exceptional ability in this area and I only regret that you have not been kept properly informed on the timing of the various requirements for PhD degree. From the point of view of the Foundation, we feel that you have performed at exceptional level and we have every confidence in your ability (Arnold, 10 mar. 1960, p.1).

That was not an isolated case. Because of two other institutional confusions, the director of the fellowships program was furious with the main persons involved at the Teachers College and wanted to remove the five nurses maintained there. As this could penalize those students, he thought of keeping them, but not sending other fellowship holders to that institution (Arnold, 23 jun. 1960, p.1).

Before finishing her fellowship, Maria Tito de Morais travelled to Los Angeles and San Francisco, in California, Portland, Seattle and New York to visit nursing schools and programs, until leaving, in August 31, 1960. This was a common practice among fellowship holders and even among people who made short trips funded by the philanthropic agency in previous years (Batista, Ferreira, 2021; Batista, 2020c). What was not expected was that Maria Tito de Morais would be immediately designated for a post in Alexandria, linked to WHO, without chances of integrating the Rockefeller staff. The philanthropic institution’s plans were dismantled, together with the investment of two years on a nurse whom they wished to have in their staff, even if, for all purposes, she had been trained to work at another organization.

This fact made Virginia Arnold and Robert Watson appalled; Ernani Braga then wrote from Brazil to Marcolino Candau, requesting the professional’s cession, from WHO, to the Rockefeller Foundation. According to Braga, he wished to take Maria Tito de Morais to the nursing section of the North American philanthropic agency, at that moment having only Arnold in charge, paying her wages and costs according to the tasks she would perform (Braga, 29 jul. 1960). Besides Ernani Braga’s letter, Robert Watson talked to a friend from WHO that was going to meet Marcolino Candau in Europe, explained the situation and asked for an intervention to his favor. However, he believed that the nurse would have to serve in Alexandria, and only then be liberated (Watson, 2 ago. 1960). All the possibilities were attempted to articulate Maria Tito de Morais’ cession, with no success.

In an evaluation of the mistakes made in this process, Virginia Arnold affirmed that the Rockefeller Foundation’s staff failed in relation to the fellowship holder for two reasons: it delayed the decision-making on continuing with her contract; and Lyle Creelman, head of the WHO nursing section and Elizabeth Hill, his assistant, blocked the efforts made to have the nurse in their staff. According to Arnold, it was a known fact that Creelman did not like the Portuguese nurse and had already prevented her on other moments, when interesting tasks were available or there were promotion opportunities. Finally, she heard of a letter from Marcolino Candau (that she did not read because she knew no Portuguese), in which he expresses reluctance to cede Maria Tito de Morais, because WHO usually dealt with governments, and not with private organizations, such as the Rockefeller. Arnold affirmed that she would not give up on that matter (Clipped..., 15 set. 1960).

On November 21, 1960, the Rockefeller Foundation registered that Maria Tito de Morais’ doctorate work would be finalized and that an advanced seminar would be held after the conclusion. She promoted significant changes as director of the High Institute of Nursing in Alexandria; was designated deputy head of the Nursing Section of WHO in 1962, and in 1966 became consultant nurse at the Regional Office of WHO in Europe: “Notes that your superior performance and long experience with WHO will be a tremendous asset to nurses and the nursing profession in the region covered by your office” (Fellowship card, s.d.). She was not incorporated into the Rockefeller staff in the rest of her professional life.

## Final considerations

The analysis of trajectories is an interesting possibility to understand fissures in norms and limitations in the performance of international agencies. The trajectories performed by participants of the Fellowship Program of the Rockefeller Foundation provide one of those opportunities, since in the local, national and international contexts, on many moments, they were opposed to what the institution proposed.

The beginning of the fellowships policy, at the end of the 1910s, was marked by a disorderly process, in which the selection of candidates did not necessarily comply with the criteria imposed. The expectation was that the best professionals would be sent, following the norms established by the institution, including in relation to research themes. However, recent historiographic analyses show that the offer of personnel was higher than the demand, and the institution’s attempts to prevent political indications of fellowship holders were many times unsuccessful, considering that not always the better qualified professionals were sent and, even with the priority of themes that directed the Rockefeller’s work, there were fellows that achieved negotiating or avoiding determinations, following itineraries and studying themes of their wish (Batista, Ferreira, 2021; Batista, 2020a, 2020b, 2019; Batista, Silva, 2020).

In 1924, the International Health Board (IHB) produced the document *Informations concerning fellowships awarded by International Health Board of The Rockefeller Foundation* (MHFMUSP, 1924), on which it attempted to formally establish criteria for the selection of fellowship holders in public health. Among many determinations, there was the search for candidates with high professional and scientific qualification, besides qualities of leadership, age preferably below 35 years old, and guarantee that the candidate would receive an appropriate job after the conclusion of the fellowship. There was the implementation of forms and standard procedures, values for single and married fellows, types of expenses covered by the agency, making clear what expenses relating to spouses would not be paid for. Finally, it made explicit aspects regarding procedures, reception, housing and moving around the study place, among others.

Although these criteria were proposed with universal validity, the case of Maria Tito de Morais demonstrates the specificity of the Portuguese context in relation to the norms for fellowship granting. The rural character, low literacy and hindrances to women’s access to education imposed challenges to the philanthropic agency, which needed to construct strategies that would enable the training of nurses. This is manifested, e.g., in the observation of candidates to nursing at the health care spaces in Lisbon, when they had never had contact with this field in their trajectories. Sending them to Toronto, since they could not be certified at the Western Reserve University, also configures a movement to ensure formal documents that would testify their education.

The Salazar regime opposed the parents of those women, which caused concern to the Rockefeller Foundation staff regarding their effective hiring by the public service. The expected rupture did not occur at that moment, and the few nurses with a diploma in Portugal worked at the two main health institutions dedicated to their education. However, Maria Monjardino abdicated the professional life to get married, and Maria Tito de Morais’ political stand resulted in the prohibition to work at the Health Center and the School of Nursing, producing a rupture with the Rockefeller Foundation’s expectations about the reproduction of the knowledge she had acquired at her international education in order to prepare new Portuguese nurses. In a way, the “key-element” called Maria Tito de Morais would not fulfil her main function due to local political conflicts.

Her qualifications and competencies resulted in a contract with WHO and the transit through various countries, enabling her to aggregate a broad experience in the field of nursing practice in a global perspective. The wish to ascend in the career implied the grant of a second fellowship. Although the Rockefeller Foundation trained Maria Tito de Morais with the justification that she would return to WHO, there was a silent movement for her to assume a position next to Virginia Arnold, in the nursing section of the international philanthropic agency. However, WHO did not cede the nurse, leaving the Rockefeller’s staff members in distress. All the investment made, thinking of its return to the Foundation, was directed to the multilateral agency from where the fellowship holder originated, in a clear demonstration that the trajectory of the Rockefeller Foundation’s fellows did not belong to it.

In this entire process, the nurse demonstrated her efforts to achieve the objectives, even if at many moments she did not trust her own potential. Proposing to herself a different path from the one taken by Portuguese women, especially during the first grant, also had its price, fears, and lack of self-confidence. Although Maria Tito de Morais was not fully aware of it, she was under dispute by two international organizations, which reveals the competence of women who acted incisively in the construction of the international knowledge on health.

## NOTES

1 The First Portuguese Republic started on October 5, 1910, with the revolution against the country’s monarchy, and lasted until a coup on May 28, 1926, when the dictatorship was implemented and later transformed into Estado Novo, between 1933 and 1974.

2 José Alberto de Faria was born in Lisbon, on August 13, 1888. He graduated from the Escola de Medicina Médico-Cirúrgica de Lisboa, in 1911, with the thesis *Tuberculina no uso clínico*. He started as a young man in the public health services. He became sub-delegate of Public Health of Lisbon in 1914 and was called to serve at the Directorate-General of Health in 1916, after the emergence of the bubonic plague. On October 12, 1926, he was designated Chief Inspector of Epidemics and Prophylaxis of Infectious Diseases. He reached the position of Director General of Health on December 20, 1928, due to Ricardo Jorge’s retirement.

3 There were different names since its creation: International Health Commission (IHC), from 1913 to 1916; International Health Board (IHB), from 1916 to 1927; and, from 1927 until its closure, in 1951, it was named International Health Division (IHD).

4 Strode graduated in medicine from the University of Pennsylvania and entered the Health Council in 1920, after having served in the war, in France. After three years as head of the Directorate of Health of Brazil (Diretoria de Saúde do Brasil), he directed the International Health Division (IHD) office in Paris, until the Second World War. Described as “balanced with common sense”, he was a manager, instead of a laboratory man.

5 Francisco Soares Branco Gentil was born in Alcácer do Sal (Portugal), in 1878, and died in Lisbon, in 1964. He worked at the Portuguese Oncology Institute (Instituto Português de Oncologia), with the position of president of the Directive Commission, from 1923 to 1961. He conceived the creation of a university hospital and the introduction of scientific nursing education, in 1940.

6 Robert Watson was the manager of the Rockefeller Foundation office in Rio de Janeiro in the 1950s.
